# Disparities in Advance Care Planning: Gaps in Clinical Notes on Comfort VS. Life-Sustaining Treatment Preferences

**DOI:** 10.1093/geroni/igaf122.3571

**Published:** 2025-12-31

**Authors:** Yuwen Ji, Alaa Albashayreh, Stephanie Gilbertson-White

**Affiliations:** University of Iowa, Iowa City, Iowa, United States; University of Iowa, Iowa City, Iowa, United States; University of Iowa, University of Iowa, Iowa, United States

## Abstract

**Background:**

Goal-concordant care is essential, yet disparities in advance care planning (ACP) persist. Electronic health record (EHRs) documentation of specific preferences, such as comfort and life-sustaining treatments (LST) and natural language processing (NLP) to extract free-text data, provides real-world information to explore patterns.

**Objective:**

To identify predictors associated with documentation of specific preferences in a cohort of older adults with chronic conditions.

**Methods:**

In a dataset of records from 14,729 older adults with heart failure, cancer, or dementia from a large academic medical center, we used a validated NLP model, Priorities-BERT (accuracy= 90.91%), to categorize documented care preferences from 3.6 million EHR notes into four groups: comfort-only, LST-only, mixed, or none. Logistic regression identified factors associated with each preference category (vs. no preference).

**Results:**

The mean age of the sample was 78.67 years (SD = 6.05). Female patients (vs. male) (OR = 1.11, 95% CI: 1.04–1.19), heart failure (OR =1.59, 95% CI: 1.34–1.89), dementia (OR = 1.33, 95%CI: 1.25-1.43), were associated with higher odds of having any priorities of care documented. Increasing age was associated with lower odds of LST (OR = 0.94, 95%CI: 0.94-0.95). Patients with heart failure (OR = 1.50, 95%CI: 1.39-1.61) or dementia (OR = 1.56, 95% CI: 1.42-1.71) were more likely to have comfort preferences documented. Conversely, cancer patients were more likely to have LST documented (OR = 1.12, 95%CI: 1.03-1.21).

**Conclusion:**

Significant disparities existed across these three diagnoses in documentation ACP preferences. Targeted clinical and health-system interventions are needed to promote equitable and timely ACP conversations for patients likely to benefit from this care.

